# When to start antiretroviral therapy: as soon as possible

**DOI:** 10.1186/1741-7015-11-147

**Published:** 2013-06-14

**Authors:** Ricardo A Franco, Michael S Saag

**Affiliations:** 1 Department of Medicine, Division of Infectious Diseases, University of Alabama at Birmingham, 1900 University Boulevard, THT 229, Birmingham, AL, 35294, USA; 2 Jim Straley Chair in AIDS Research, Center for AIDS Research, University of Alabama at Birmingham, 845 19th Street South, BBRB 256, Birmingham, AL 35294-2170, USA

**Keywords:** HIV infection, CD4 lymphocyte count, When to start, Antiretroviral therapy, Early treatment

## Abstract

**Background:**

The debate regarding ‘When to Start’ antiretroviral therapy has raged since the introduction of zidovudine in 1987. Based on the entry criteria for the original Burroughs Wellcome 002 study, the field has been anchored to CD4 cell counts as the prime metric to indicate treatment initiation for asymptomatic individuals infected with Human Immunodeficiency Virus. The pendulum has swung back and forth based mostly on the relative efficacy, toxicity and convenience of available regimens.

**Discussion:**

In today’s world, several factors have converged that compel us to initiate therapy as soon as possible: 1) The biology of viral replication (1 to 10 billion viruses per day) strongly suggests that we should be starting early. 2) Resultant inflammation from unchecked replication is associated with earlier onset of multiple co-morbid conditions. 3) The medications available today are more efficacious and less toxic than years past. 4) Clinical trials have demonstrated benefits for all but the highest CD4 strata (>500 cells/μl). 5) Some cohort studies have demonstrated the clear benefit of antiretroviral therapy at any CD4 count and no cohort studies have demonstrated that early therapy is more detrimental than late therapy at the population level. 6) In addition to the demonstrated and inferred benefits to the individual patient, we now have evidence of a Public Health benefit from earlier intervention: treatment is prevention.

**Summary:**

From a practical, common sense perspective we are talking about life-long therapy. Whether we start at a CD4 count of 732 cells/μl or 493 cells/μl, the patient will be on therapy for over 40 to 50 years. There does not seem to be much benefit in waiting and there likely is significant long-term harm. Do not wait. Treat early.

The counter-argument to this debate topic can be freely accessed here: http://www.biomedcentral.com/1741-7015/11/148.

## Background

“*All Scientific work is incomplete - whether it be observational or experimental. All scientific work is liable to be upset or modified by advancing knowledge. That does not confer upon us a freedom to ignore the knowledge we already have, or to postpone the action that it appears to demand at a given time.*”

*- Sir Austin Bradford Hill*[1].

In 1986 zidovudine (AZT) had a striking efficacy in decreasing mortality among patients with Human Immunodeficiency Virus (HIV) infection and advanced Acquired Immunodeficiency Syndrome (AIDS). Those patients had very low CD4 T cell counts, profound immunodeficiency and a very high risk of developing opportunistic infections (OIs), especially *Pneumocystis jirovecii* pneumonia. After 24 weeks of treatment, 19 placebo recipients and 1 AZT recipient died (*P* <0.001). This remarkable benefit of AZT led to early discontinuation of the placebo-based, first successful HIV treatment trial [2]. These compelling results soon made investigators interested in the potential benefits of treatment at earlier stages of the disease, prior to the development of OIs or drops in CD4 T cell counts to below 200 cells/μl [3]. However, subsequent trials of AZT monotherapy in patients with early infection failed to show evidence of durable benefit in halting the progression of the disease and longer survival [3-5]. The reverse transcriptase inhibitors that followed AZT in the early 1990s - didanosine, zalcitabine, and stavudine - also were relatively weak antiretroviral agents that at best lowered the viral load by 0.7 log_10_ copies/ml and their individual use was followed by breakthrough HIV viremia with resistant virus [6,7].

Over subsequent years substantial progress was made in developing more potent antiretroviral agents and regimens. Novel inhibitors of the HIV protease, such as ritonavir and indinavir, were able to lower plasma viremia by 2.0 log_10_ copies/ml and certain non-nucleoside blockers of the reverse transcriptase, such as nevirapine, exhibited inhibitory effect of 1.0 to 1.5 log_10_ copies/ml [8,9]. Combination therapy, which came into vogue in the early 1990s, of zidovudine plus lamivudine had shown a promising activity of about 1.7 log_10_ copies/ml *in vivo*[7]. The advent of highly active antiretroviral therapy (HAART) by the mid-1990s brought new hopes for the advocates of the “hit early, hit hard” approach [10]. However, subsequent studies analyzing patient cohorts on earlier HAART regimens would still show no difference in HIV-related complications or mortality comparing early initiation of HAART (CD4 T cell counts ≥350 cells/μl) versus delayed HAART initiation (CD4 cell counts of 200 to 350 cells/μl) [11,12]. The cumulative toxicities and poor tolerability of initial HAART regimens, the negative impact of pre-existent HIV resistance among those treated with inadequate regimens years before HAART, and the need for life-long treatment made clinicians and guidelines eventually move away from this strategy [13,14].

The debate regarding optimal time to initiate antiretroviral (ARV) therapy has continued since the early years of HAART [15,16] and remains active in the current stage of therapies against HIV [17]. Herein, we enumerate several reasons why HIV should be treated as early as possible in today’s world. Arguments to the contrary are outlined in a debate article published in *BMC Medicine*[18].

## Discussion

### The biology

During AZT monotherapy, an 80% reduction in viral load (0.9 log_10_ copies/ml) was noted as soon as one week after initiation of therapy followed by a fast, nearly symmetric return to baseline levels within one week after treatment discontinuation [19]. Subsequent viral dynamics studies using more potent HIV protease and reverse transcriptase inhibitors showed how fast rounds of *de novo* virus infection occur, making 1 to 10 billion new viral copies per day [9,20]. With this magnitude of replication, it was estimated that both the viral life cycle and the half-life of infected CD4 T cells were as short as one day or less with several million CD4 T cells being infected each day [20]. These findings had a deep impact on the understanding of how destruction of the immune system occurs and why CD4 counts decline over time, even during the period of “clinical latency” [9]. Based on the above, it has been reasonable to consider that early and profound suppression of the HIV replication brings several benefits: it reduces the high levels of ongoing inflammation, creates a higher virologic hurdle for its emergence and preserves the immune system integrity before there is loss of vital clones of responsive cells [21]. Indeed, the biology strongly suggests that inhibition of relentless cycles of viral replication should be accomplished as soon as possible.

### The association of inflammation and disease

The breakthroughs in understanding HIV pathogenesis fueled subsequent research beyond the boundaries of unchecked viral replication. Uninterrupted CD4 T cell activation and apoptosis are the hallmarks of both HIV disease progression as well as the basis of a persistent inflammatory state, which is associated with deleterious cardiovascular and metabolic consequences to the host [22]. Although the reduction of T cell activation (and inflammation) brought by effective therapy never reaches ‘normal’ levels (as measured in uninfected controls) [23], early therapy substantially reduces residual T cell activation compared with that in subjects not on therapy [24]. Even though a cause-effect relationship is unproven at this point, elevated inflammatory biomarkers, such as D-dimer, C-reactive protein, hyaluronic acid, and soluble CD14, all correlate with the risk for all-cause mortality among infected subjects [25-29]. This association adds further momentum to initiation of ARV therapy earlier to minimize the duration of exposure to high levels of inflammation [23].

Schouten and colleagues have recently shown that the prevalence of non-AIDS comorbidities in HIV-infected adults aged 50 to 55 years was comparable to uninfected adults older than 65 years of age. This verified earlier onset of comorbid conditions occurred despite having 84% of the HIV-infected patients with undetectable viral loads and remained consistent even after controlling for factors such as age, gender and smoking [30]. Despite intrinsic ascertainment bias in studies of this kind, these findings highlight the potential role of exposure to inflammation on non-AIDS comorbidities, which are the leading cause of death in HIV-infected patients today. It also provides a “sense of urgency” in the initiation of treatment. The mean CD4 count nadir in the study was 330 cells/μl and the mean CD4 count the year prior to enrollment was 548 cells/μl. Considering that the mean length of infection prior to treatment initiation was 11 years, it seems that this population received treatment relatively early, but not nearly enough in order to have outcomes comparable to uninfected controls. In the Veterans Aging Cohort Study (VACS), Althoff and colleagues observed an 81%, 43% and 84% increase in adjusted incidence of myocardial infarction (MI), end-stage renal disease (ESRD) and AIDS-related cancers (lung, liver, anal, oropharyngeal cancers and Hodgkins lymphoma). Age of diagnosis did not differ between the HIV-positive and HIV-negative groups, providing little evidence for the concept of premature aging. Nevertheless, the much higher incidence of hard outcomes, such as MI, ESRD and cancer, is complementary to the hypothesis of earlier development of pre-morbid conditions in HIV [31]. Moreover, in a study by van Sighem and colleagues, a cohort of 13,077 people diagnosed with HIV in 1998 or later was analyzed. In this treatment-naïve population, those who initiated therapy with CD4 counts below 200 more than quadrupled the risk of the composite non-AIDS endpoints (major cardiovascular diseases, liver cirrhosis and non-AIDS malignancies) than those who initiated treatment with CD4 counts >500 cells/μl. Counts between 200 and 349 cells/μl were associated with a more than doubled risk, and risks for those with counts between 350 and 499 cells/μl were not significantly different from those with >500 cells/μl, though there was a trend toward higher risk of the composite endpoint (RR 1.23, CI 0.85 to 1.78) [32]. Taken together, these data show that ongoing chronic inflammation is a potential driving force behind morbidity and mortality, a finding that has been cited as one of the major scientific insights of the past decade [33].

### Better tolerated medications today

Newer medications and formulations have addressed many of the limitations of earlier regimens in terms of short and long-term antiretroviral toxicities. Therapies against HIV have become easier to administer, less toxic and more potent. Undoubtedly, concerns about tenofovir-associated kidney dysfunction, bone demineralization and potential increases in cardiovascular disease risk remain [34-37]. Nevertheless, newer protease inhibitors have been associated with far fewer adverse effects, such as dyslipidemia, insulin resistance and gastrointestinal intolerance [38,39]. Likewise, newer nucleoside reverse-transcriptase inhibitors have virtually no associated lipodystrophy or major mitochondrial dysfunction [40]. Novel HIV treatments are able now to offer more convenient dosing. Fixed-dose combination options administered once daily have led to more uniformity in initial antiretroviral therapy. In assessing prescribing practices in our clinic, the most dramatic shift in drug selection involved the incremental use of emtricitabine plus tenofovir plus efavirenz, from 0% in 2003 to 85% in 2007. This reflected better acceptance of a simpler regimen that can be administered as a single, daily pill [41]. These dramatic advances have tremendously impacted clinical practice and compelled clinicians and investigators to revisit the question of the ideal time to initiate therapy, weighing the relative risks and benefits.

### Cohort data

Clinical research has continued to evolve, extracting evidence from contemporary clinical practice. Data from the North America-AIDS Cohort Collaboration on Research and Design (NA-ACCORD) clearly demonstrated that the adjusted mortality rates were statistically higher among the 6,935 patients who deferred therapy until their CD4 counts fell to <500 cells/μl than in the 2,200 patients who started therapy with CD4 counts >500 cells/μl (risk ratio: 1.94, 95% CI: 1.37 to 2.79). However, the absolute risk of death was low in both groups: 5.1% in the deferred therapy and 2.9% in the early therapy group [42]. Although large and representative of the HIV-infected patients in care in the United States, the study had limitations intrinsic to its retrospective design, including the relatively small number of deaths and the potential for unmeasured confounders that might have influenced outcomes independent of treatment. Indeed, two other large cohort studies, the Antiretroviral Therapy-Cohort Collaborative (ART-CC) and the Concerted Action on SeroConversion to AIDS and Death in Europe (CASCADE) collaboration, did not identify a benefit of earlier initiation of therapy in reducing AIDS progression or death [43,44]. These studies, however, shared the same “limitations” of the NA-ACCORD study, with a fortunate low proportion of treated patients progressing to AIDS or death during follow-up. The ART-CC study also was limited by the period of observation beginning with initiation of therapy. Of note, no cohort study to date has demonstrated any clear evidence of greater harm among those initiating therapy with CD4 counts >500 cells/μl. To the contrary, most have shown trends toward benefit but, owing to smaller numbers of patients in the >500 cells/μl group and the relative absence of mortality events, did not demonstrate statistical benefit (with the notable exception of the NA-ACCORD study that did show statistical benefit).

Due to these intrinsic limitations of cohort studies in analyzing rare outcomes, investigators have tried different methods and designs in evaluating the evidence behind early treatment. A recent clinical trial (Setpoint Study) randomly assigned patients, who were within six months of HIV seroconversion, to receive either immediate treatment for 36 weeks or deferred treatment (when CD4 counts were <350 cells/μl). More than 57% of the study participants had CD4 counts >500 cells/μl. The deferred treatment group had a statistically higher risk of meeting treatment initiation criteria (for example, CD4 <350 cells/μl) resulting in earlier discontinuation of the study. While this study was not a clinical endpoint study, these results illustrated that the time from diagnosis of early infection to the need for initiation of therapy was shorter than anticipated [45].

Other data support earlier initiation of treatment. Chronically infected patients delaying therapy until CD4 T cells ≤350 cells/μl have suboptimal CD4 T cell count recovery. After six years of ARV therapy, those who delayed therapy reached a CD4 count plateau below 500 cells/μl, which was significantly lower than patients starting therapy earlier [46].

The benefits of early treatment go beyond hard immunologic parameters and translate into a higher likelihood of overall treatment success. The implementation of universal treatment of all HIV-infected persons in a large, publicly-funded clinic in San Francisco in 2010 led to a six-fold increase in the probability of viral suppression. In 534 patients entering the clinic with CD4 counts >500 cells/μl, the one-year incidence of viral suppression increased from 14% to >52% after adopting the approach [47]. These results are complimentary to data from another large outpatient cohort showing that major resistance mutations were 50% less likely in patients starting therapy with CD4 count >350 cells/μl versus <200 cells/μl despite greater treatment exposure [48]. These data run counter to the ‘wait until later’ proponents who predicted that resistance would be more common among those who started therapy earlier. It is not more common; it is less. Given the concerning reality of the low proportions of treatment success in real life settings such as above, and the compared success proportions often greater than 80% in clinical trials, early treatment must be accompanied by excellent individual care in order to ensure the highest therapy adherence.

As we start to adopt the “test and treat” approach, data from the Johns Hopkins HIV clinic have demonstrated that starting therapy earlier is a cost-effective strategy by the generally accepted benchmark in the US [49].

### Guidelines

**Figure 1:**
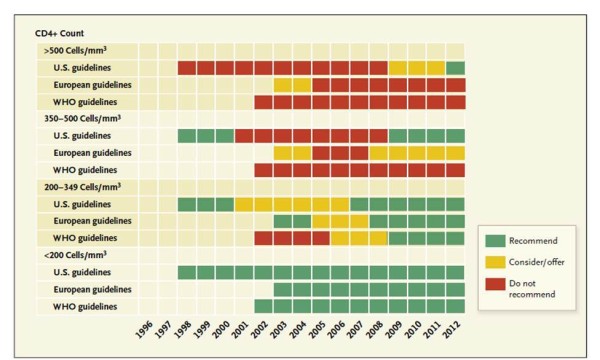
**When to start HAART among asymptomatic HIV + patients according to guidelines: 1998 to 2012. **Criteria from the United States are derived from the IAS-USA Treatment Guidelines and the Department of Health and Human Services Guidelines for Antiretroviral Therapy for Adults and Adolescents; European guidelines are derived from the European AIDS Clinical Society (EACS) Guidelines; and the World Health Organization (WHO) Guidelines are from the WHO Antiretroviral Therapy Guidelines for Adults and Adolescents. Adapted with permission from Dr. Marco Vitoria, MD of the World Health Organization and the Massachusetts Medical Society/New England Journal of Medicine [50].

Many of the observational cohort studies have supported the earlier initiation of HAART, resulting in a renewed confidence among many guideline committees to recommend initiation of therapy for those with higher CD4 counts in resource rich countries (see Figure 1). Yet, guidelines for starting therapy for those in resource-limited settings typically recommend starting therapy later in the course of infection [50]. Ideally, no difference should exist between guideline recommendations for when to start treatment solely based on resources. Rather, the biologic evidence of when to start is very likely the same regardless of location. However, resources often may dictate what is implementable or not in a given location. “Guidelines” are simply guidelines, not directives or imperatives. They represent the ideal and it is up to the local Ministries to decide what is feasible in each location at any moment in time.

### Public health

In addition to potential gains in viral load suppression for the individual, in terms of improved outcomes and resistance mitigation, the reduction in viral load substantially reduces new HIV infections at the community level and, therefore, is extremely important from the standpoint of public health. The results of the HIV Prevention Trials Network (HPTN 052) proved this benefit unequivocally. This was a multi-continental trial that enrolled 1,763 HIV-serodiscordant couples comparing immediate treatment versus delayed therapy for the HIV-infected partner [51]. At study entry, 98% of the participants were in heterosexual, monogamous relationships and were counseled on behavioral modification and condom use. Twenty-eight linked HIV transmission events were identified during the study period, but only one event occurred in the early therapy arm (and this transmission occurred early into treatment before viral load was fully suppressed). This 96% reduction in transmission associated with early ART was highly significant (hazard ratio (HR) 0.04; 95% confidence interval (CI): 0.01 to 0.27, *P* < 0.001). These results conveyed the message that early therapy is more effective in preventing transmission of HIV than all other behavioral and biomedical prevention interventions studied to date, including condom use, male circumcision, vaginal microbicides, HIV vaccination and pre-exposure prophylaxis.

Other observational studies and modeling analyses have provided similar conclusions as it relates to a decreased rate of HIV transmission in serodiscordant heterosexual couples following the introduction of ART [52-57]. In the United States, 25% of the infected adults are unaware of their status and are responsible for >55% of new infections [58]. HIV treatment is prevention. Although public health interests should not be a primary reason for early treatment initiation, these data do provide further rationale for earlier initiation of treatment.

### Clinical trial data

Despite the high-quality, cohort-derived evidence and the balance in favor of early treatment, some investigators remain skeptical and reluctant to adopt this strategy due to the lack of clinical trials of early versus delayed therapy for those with CD4 cell counts >500 cells/μl. The Strategic Timing of Anti-Retroviral Treatment (START) trial has been in enrollment phase and is expected to provide the first randomized trial evidence of whether immediate initiation of treatment in patients with CD4 cell counts greater than 500 cells/μl is superior to delaying initiation of HAART until the CD4 cell count falls below 350 cells/μl [59]. Although clinical trials are touted as the study modality capable of providing the strongest evidence in guiding clinical practice, they are not necessarily the right modality to answer all clinical questions. In the case of ‘when to start’, several confounders and limitations exist. First, the time to development of complications of either viral infection or drug toxicity in these subjects who have relatively early HIV disease is quite long. Therefore, a three-year study endpoint likely is too soon to answer the question. Rather, the impact of ongoing, unchecked viral replication likely will not become evident until many years later. Findings similar to those described in the van Sighem study, as outlined above [32], likely will emerge during the early results of the START study; that is, trends toward supporting the higher CD4 count group that will require several years of further follow-up.

Second, although clinical trials have the advantage of randomization, there still are inherent limitations in study eligibility criteria and study referral patterns that can limit the generalizability of the findings. In particular, the patients referred to this study will be only those deemed to have clinical equipoise regarding when to start treatment. Those who the clinicians either do not want to treat now (poor treatment candidates or those who are not willing to start treatment) or who they do want to treat now (patients who need immediate treatment and cannot wait for routine study procedures, including extra time for randomization) will not be referred to the study. This could represent up to three quarters of potentially eligible patients. So, in essence, some of the same ‘channeling biases’ present in clinical practice and, therefore, cohort studies, are at play among those individuals referred (or not referred) to a clinical trial. If the question was whether to ever treat HIV, a clinical trial seems like a great approach to answer the question. In the case of defining subtle differences within a narrow window of time, the results do not seem worth the efforts and/or the costs.

### Common sense

The decision to start early treatment is an event that occurs in a relatively small window of time in the life span of a person with HIV infection. While some patients have stable CD4 counts over time (for example, “Elite Controllers”), the majority experience drops in CD4 counts of 40 to 80 cells/μl/year. As an example, over as few as two years, and on average five years, CD4 cell counts can drop from 500 cells/μl to 350 cells/μl. Five extra years of therapy out of a total of 40 to 50 years on treatment for those living a near-normal life span (for example, treatment from age 25 years to 75 years) represents relatively minor differences in long-term exposure to treatment. However, those five extra years of continued exposure to unchecked viral replication represent potential substantial harm as demonstrated by the known biology of the infection. Rather than the ‘feared’ unnecessary exposure to drugs for only a fraction of a person’s lifetime, the likely harm comes from relentless replication of HIV, inflammation, destruction of lymphoid tissue, likely increased cardiovascular events, higher rates of certain malignancies and accelerated cognitive decline.

## Summary

In conclusion, the balance of available data strongly supports starting treatment in nearly all individuals regardless of CD4 T cell counts. Early treatment recommendations are based on our understanding of HIV biology, HIV pathogenesis, the availability of better drugs, the evidence from cohort studies, and the public health implications of viral load suppression and decreased transmission. Exceptions might be among the very small population of individuals who are ‘elite controllers,’ defined as those who have undetectable virus in the absence of antiretroviral therapy. For everyone else, to wait on randomized clinical trial data could well be doing harm. The time spent waiting is time that the patients cannot get back and the long-term damage associated with waiting could well be irreversible.

Just prior to the quote referenced in the Background section above, Sir Austin Bradford Hill had stated:

“*In asking for very strong evidence I would, however, repeat emphatically that this does not imply crossing every ‘t’, and swords with every critic, before we act”*[1]*.*

Until proven otherwise, we should heed Sir Austin Bradford Hill’s admonition and act upon the evidence we have in hand, which overwhelmingly tells us to treat early. What are we waiting for?

## Abbreviations

ART-CC: Antiretroviral Therapy-Cohort Collaborative; ARV: Antiretroviral; AZT: Zidovudine; CASCADE: Concerted Action on SeroConversion to AIDS and Death in Europe; CD4 count: A measure of the number of helper T cells per cubic millimeter of blood, used to analyze the prognosis of patients infected with HIV; CI: Confidence interval; EACS: European AIDS Clinical Society; ESRD: End-stage renal disease; HAART: Highly active antiretroviral therapy; HPTN: HIV Prevention Trials Network; HR: hazard ratio; MI: Myocardial infarction; NA-ACCORD: North America-AIDS Cohort Collaboration on Research and Design; OIs: Opportunistic infections; RR: Relative risk; START: Strategic Timing of Anti-Retroviral Treatment; VACS: Veterans Aging Cohort Study; WHO: World Health Organization.

## Competing interests

RF has no competing interests. MSS is a consultant for BMS, Gilead, Merck, ViiV and Janssen. He has received research support from Abbvie, BMS, BI, Gilead, GSK, Merck, ViiV and Janssen.

## Authors' information

Dr. Franco is an Associate in Infectious Diseases at UAB. Dr. Saag is Professor of Medicine and Director of the UAB Center for AIDS Research. This work was presented, in part, at the “HIV 11” meeting in Glasgow, Scotland, November 2012.

## Pre-publication history

The pre-publication history for this paper can be accessed here:

http://www.biomedcentral.com/1741-7015/11/147/prepub

## Note

This article is part of the cross journal collection HIV 30 years on. Other articles in this series can be found at http://www.biomedcentral.com/series/HIV_30.
